# Greater Glycemic Burden Is Associated with Further Poorer Glycemic Control in Newly-Diagnosed Type 2 Diabetes Mellitus Patients

**DOI:** 10.3390/nu14020320

**Published:** 2022-01-13

**Authors:** Wei-Lun Wen, Hui-Chun Huang, Hsiu-Chu Lin, Wan-Ching Lo, Szu-Chia Chen, Mei-Yueh Lee

**Affiliations:** 1 Department of Internal Medicine, Lee’s Endocrinology Clinic, Pingtung City 900, Taiwan; stevenwen760829@gmail.com; 2 Department of Internal Medicine, Kaohsiung Municipal Siaogang Hospital, Kaohsiung Medical University, Kaohsiung 812, Taiwan; 3 Division of Endocrinology and Metabolism, Department of Internal Medicine, Kaohsiung Medical University Hospital, Kaohsiung 807, Taiwan; 4 Department of Electronics Engineering, Institute of Electronics, National Chiao Tung University, Hsinchu 300, Taiwan; harlanhaung@gmail.com; 5 Hengchun Navaids Site, Kaohsiung Aviation Facilities Sector, Air Navigation and Weather Services, Ministry of Transportation and Communications, Hengchun 946, Taiwan; 6 Certified Diabetic Educator of Endocrinology & Metabolism Ward, Kaohsiung Medical University Hospital, Kaohsiung 807, Taiwan; 830323@ms.kmuh.org.tw; 7 Department of Nursing, Kaohsiung Municipal Siaogang Hospital, Kaohsiung 812, Taiwan; 860164@kmhk.org.tw; 8 Division of Nephrology, Department of Internal Medicine, Kaohsiung Medical University Hospital, Kaohsiung Medical University, Kaohsiung 807, Taiwan; 9 Research Center for Environmental Medicine, Kaohsiung Medical University, Kaohsiung 807, Taiwan; 10 Faculty of Medicine, College of Medicine, Kaohsiung Medical University, Kaohsiung 807, Taiwan

**Keywords:** glucotoxicity, glycemic burden, glycemic control, glycemic durability

## Abstract

**Aims:** hyperglycemia impairs pancreatic β-cell function instantly, also known as glucotoxicity. It is unknown whether this insult is temporary or sustained, and little real-world evidence needs to reflect the relationship between hyperglycemic burden, per se, and glycemic durability. **Materials and Methods:** a retrospective observational cohort study was conducted to recruit newly-diagnosed type 2 diabetes mellitus (T2DM) patients. Durability was defined as the episode from first glycated hemoglobin A1c (HbA1c) below 7.0% to where it exceed 8.0% (with treatment failure) or where study ended (without treatment failure). Glycemic burden was defined with the area above a burden value line (HbA1c = 6.5%) but under the HbA1c curve (AUC), and it was then divided into two compartments with the demarcation timepoint once HbA1c was treated below or equal to 7.0%; the former AUC’ represented the initial insult; the latter AUC” represented the residual part. Multivariable regression models assessed factors associated with durability in whole participants and two distinct subgroups: patients with baseline HbA1c > 7.0% or ≤7.0%. **Results:** 1048 eligible participants were recruited and analyzed: 291 patients with treatment failure (durability 26.8 ± 21.1 months); 757 patients without treatment failure (durability 45.1 ± 31.8 months). Besides age, glycemic burden was the only constant determinant in the two subgroups. AUC’ or AUC” increased treatment failure, respectively, in baseline HbA1c > 7.0% or ≤7.0% subgroup [per 1%/90 days hazard ratio (95% confidence interval): 1.026 (1.018–1.034) and 1.128 (1.016–1.253)]. Other determinants include baseline HbA1c, initial OAD, and education level. **Conclusions:** in patients with newly-diagnosed T2DM, glycemic durability was negatively associated with greater glycemic burden.

## 1. Introduction

Glucotoxicity plays a pathogenic role in the early stages of type 2 diabetes mellitus (T2DM) as in vivo evidence reveals the loss of first-phase insulin secretion along with elevating plasma glucose [1]. In vitro, the toxicity was proved to be a continuous rather than a threshold function of glucose concentration, and the shorter the period of antecedent glucose toxicity, the more likely that full recovery of β-cell function will occur [2]. In vivo, the acute first-phase insulin response might be reversed after hyperglycemia is resolved, as T2DM remissions were found initially after bariatric surgery or intensive medication therapy in Remission Evaluation of Metabolic Interventions in Type 2 diabetes (REMIT study) [3,4]. Nevertheless, extended research found that T2DM relapsed after years follow-up [5,6]. Therefore, we are curious about whether the insult of glucotoxicity in newly-diagnosed T2DM patients are not transient but persistent, which may be further presented in the form of secondary treatment failure.

Hyperglycemia not only induces glucotoxicity but also increases microvascular complications, macrovascular complications, and associated mortality in T2DM patients [7,8,9,10]. This damage is obviously persistent and accumulative, and there is also a classical phenomenon called “legacy effect” or “metabolic memory”, which means early poorer glycemic control has durable effects, while even further glycated hemoglobin A1c (HbA1c) values converged, and the higher incidence of cardiovascular events and mortality were observed a decade later [11,12]. In fact, the underlying pathophysiology of hyperglycemia induced these complications, or glucotoxicity could be attributed to a similar upstream mechanism, that is, supraphysiological glucose concentrations drive mitochondrial reactive oxygen species (ROS) overproduction, the former merely in vascular endothelial cells and the latter in pancreatic β-cell [13,14]. ROS may induce long-lasting pathological changes through multiple pathways, including altering histone modifications, DNA modifications, expression of noncoding RNAs, and ATP-dependent chromatin remodeling in these affected cells. These epigenetic changes are supposed to pass to daughter cells with each division and cause metabolic memory [15,16]. Therefore, it seems reasonable to postulate that initial glucotoxicity, indeed, prolongs insulting pancreatic β-cells and decreases glycemic durability, which is usually defined as the maintenance of optimal glycemic control.

Maintaining T2DM patients’ glycemic durability is always a concern of clinical practice because it is difficult in many patients, and the progressive HbA1c values would increase the complications mentioned in the above paragraph [17]. Researchers have found several factors that might affect or predict glycemic durability. One of these is anti-diabetic medication use. A Diabetes Outcome Progression Trial (ADOPT) and Vildagliptin Efficacy in combination with metfoRmIn For Yearly treatment of type 2 diabetes (VERIFY) trial revealed an oral antidiabetic (OAD) medication choice could delay treatment failure [18,19]. Another important factor is the age of onset for T2DM. Compared with older patients, young-onset type 2 diabetes (YOD) are less likely to achieve glycemic control no matter if disease duration is long or short [20,21]. Finally, higher baseline HbA1c value, before starting OAD, is also an important prediction factor of poorer durability, which is presented by a higher risk of secondary failure in studies [22,23]. However, baseline HbA1c value could only represent the hyperglycemia amplitude of one timepoint but not consider the duration of hyperglycemia episodes that could damage the pancreatic β-cell.

Glycemic burden can be precisely defined with the cumulative amount by which HbA1c has exceeded a specified treatment goal or threshold, i.e., the area above a line but under the HbA1c curve (AUC) [24]. This calculation method has been verified in several studies, revealing relationships between glycemic burden and microvascular complications, macrovascular complications, or risk of cardiovascular disease hospitalization [25,26,27]. To our knowledge, little real-world evidence directly reflects the relationship between glycemic durability and hyperglycemia burden. Therefore, we undertook the current study to determine whether glycemic burden, per se, would impair long-term glycemic durability after controlling other known risk factors.

## 2. Materials and Methods

### 2.1. Study Population and Setting

**Figure 1:**
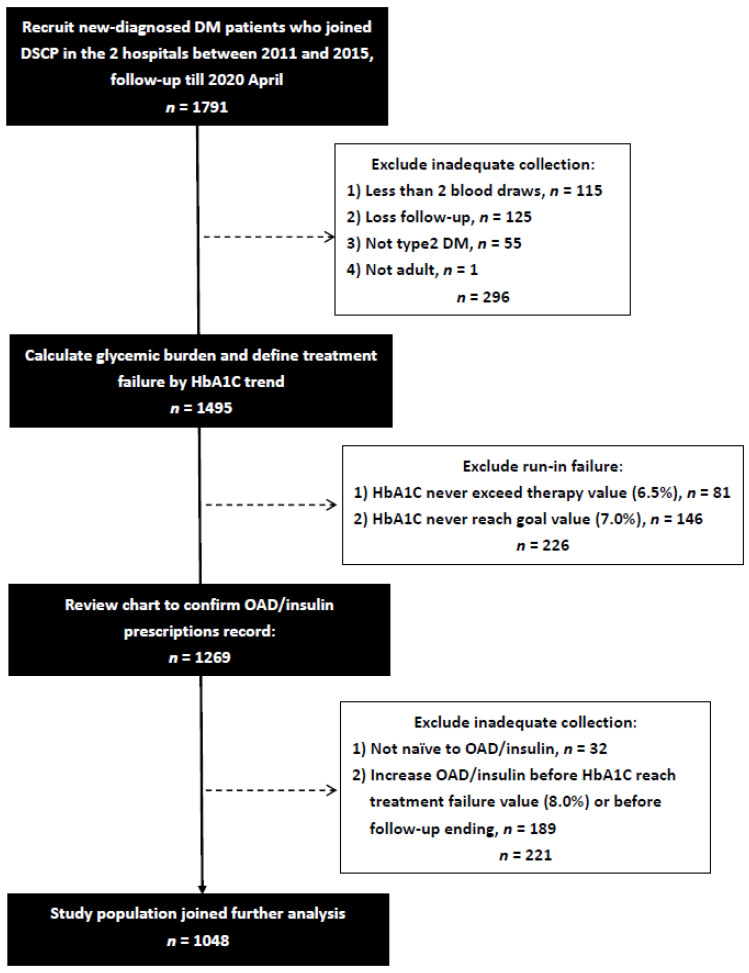
Flow chart of recruited participants.

We conducted this retrospective observational cohort study at two hospitals: one is a tertiary teaching hospital center, and the other is a regional hospital, in southern Taiwan. Newly-diagnosed diabetes mellitus patients who joined the Diabetes Shared Care Program (DSCP) [28], an integrated diabetes care model designed to increase the quality of diabetes care in Taiwan, since 1 January 2011 to 31 December 2015 were recruited. Some patients were excluded if they met any of the following criteria: (1) Less than two HbA1c blood draws, (2) Loss follow-up, which was defined with too long of an interval between last two HbA1c blood draws (>365 days) or no HbA1c blood draw after run-in period, (3) not T2DM, including prediabetes, gestational diabetes, type 1 diabetes, other types of diabetes, or undetermined, (4) non-adult, with age <18, (5) have ever been prescribed OAD or insulin, (6) HbA1c value never reaches starting treatment value (HbA1c < 6.5%). Furthermore, we excluded the patient who did not complete run-in period (initial baseline HbA1c > 7.0% but further follow-up HbA1C never decreases to 7.0%) or the patient whose prescribed OAD categories or insulin injection frequencies had increased compared to the initially prescribed before the end of stable glycemic period. A total of 1048 newly-diagnosed drug-naïve T2DM patients were enrolled after the exclusion of 739 participants due to inadequate collection or failing to complete run-in period (Figure 1). The study protocol was approved by the Institutional Review Board of Kaohsiung Medical University Hospital (number: KMUHIRB-E(I)-20200204).

### 2.2. Definition of the Glycemic Burden, Glycemic Durability, and Initially Prescribed OAD/Insulin

**Figure 2:**
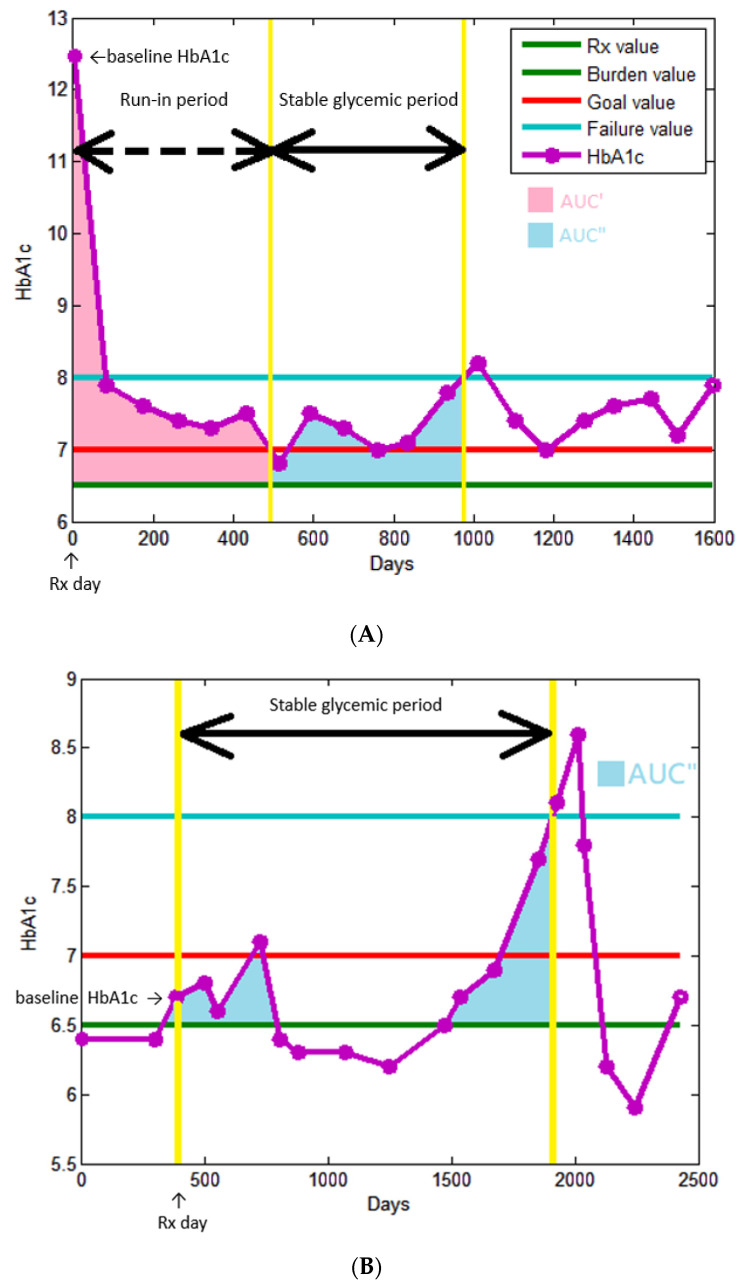
(**A**) An example of baseline HbA1C > 7.0%. (**B**) An example of baseline HbA1C ≤ 7.0%. Rx vale = starting treatment value. Goal value = treatment goal value. Failure value = treatment failure value.

Figure 2 illustrates these definitions. We defined HbA1c 6.5% as the starting treatment value, which means diabetes treatment should be started thereafter. The first HbA1c value exceed or equal to this threshold was defined as baseline HbA1c, and the blood draw date was defined as Rx day. We reviewed the electronic medical record (EMR) to confirm the initially prescribed OAD/insulin: the first prescription > 14 days at our outpatient department after Rx day, so switching drugs due to initial adverse effect would be excluded. OAD was divided into eight categories: biguanides, thiazolidinedione (TZD), dipeptidyl peptidase-4 inhibitor (DPP4i), glucagon-like peptide-1 receptor agonists (GLP1RA), sulfonylurea (SU), glinides, α-glucosidase inhibitor (AGI), and sodium-glucose cotransporter 2 inhibitor (SGLT2i). If there was no OAD prescription record in 90 days after the Rx day, this patient was classified into no OAD use subgroup. Other three subgroups: monotherapy, two combination therapy, and three or more combination therapy were classified according to the sum of numbers of initially prescribed OAD categories. The insulin prescription history was divided into three subgroups: no insulin use, basal insulin use, and basal and pre-prandial insulin use.

Besides the starting treatment value, there were three other specific HbA1c values defined in our study. Treatment goal value was defined as 7.0% to be in line with local and international treatment guidelines [29]. Treatment failure value was defined as 8.0% because, according to previous study about clinical inertia, most physicians would not adjust OAD until the difference between treatment goal value and actual HbA1c value greater than 1.0% [30]. We defined 6.5%, but not 7.0%, as burden value because increasing microvascular complications were observed in T2DM patients far lower than this threshold [31].

The major outcome of this study is glycemic durability. We divided the study observation period into two parts after the patient received treatment (Rx day). The first part is run-in period, which only existed in the patient with baseline HbA1c > 7.0%, defined as the duration between Rx day to the HbA1c decrease to treatment goal value (HbA1c = 7.0%). In conjunction with the run-in period continuously, the second part was called stable glycemic period, and it ended up, until the HbA1c rebounded to treatment failure value (HbA1c = 8.0%), in the subgroup with treatment failure or until the date of last blood draw in the subgroup without treatment failure (HbA1c always <8.0% in the second part) before the end of this study period, 30 April 2020. It is worth noting that, if the actual HbA1c blood draw declined below treatment goal value or exceeded above treatment failure value (HbA1c < 7.0% or >8.0%), we assumed linear trend between two HbA1c measurements and used linear interpolation to define the virtual date that HbA1c value reached these thresholds. Patients with baseline HbA1c ≤ 7.0% did not consist of the run-in period intrinsically and started stable glycemic period directly, and it began on Rx day and ended as the rule we just mentioned. To define the glycemic durability, we only considered the stable glycemic period and excluded the run-in period. This procedure made patients with different baseline HbA1c stand at the same starting line. Furthermore, the initial run-in period represented the temporary recovery from hyperglycemia, but what we focused on was whether further good glycemic control could last longer, which was determined by the sequential stable glycemic period.

Glycemic burden is one of the major determinants we wanted to investigate in this study, and AUC is a good surrogate marker confirmed in previous published studies [24,25,26,27]. The AUC was calculated by rectangle rule in our study instead of trapezoidal rule in the previous study [25]. Both two rules are methods to approach numerical integration of AUC: the original graphic was subdivided into many thin rectangles or thin trapezoids, so the area could be calculated by summing up all these geometric shapes. Due to the premise that the trend of HbA1c was linear between discrete time points, the AUC result would be identical whether using rectangle or trapezoidal rule [32]. Additionally, we divided AUC into two parts: AUC’ and AUC”. The former represented the burden during run-in period; the latter represented the burden during stable glycemic period. This arrangement could help to investigate whether metabolic memory also exists in the relationship between glycemic burden and glycemic durability. Furthermore, in coordination with most guidelines and clinical practices, performing HbA1c blood draw every 3 months, each unit of AUC’ and AUC” represents 90 days of an HbA1c that is one percentage point higher than the burden value (6.5%).

### 2.3. Other Covariates and Divided Subgroups 

Beside baseline HbA1c and initially prescribed OAD/insulin, the following variables were also recorded at baseline: demographics (age, sex, and education history), examination findings (systolic blood pressure (SBP), diastolic blood pressure (DBP), body mass index (BMI)), laboratory data (triglyceride (TG), total, high-density lipoprotein (HDL)- and low-density lipoprotein (LDL)-cholesterol, estimated glomerular filtration rate (eGFR), and alanine aminotransferase (ALT)). Education history was divided into 3 subgroups: elementary school or uneducated, high school, and university or higher. eGFR was calculated using simplified Modification of Diet in Renal Disease (MDRD) formula [33]. BMI was calculated as weight/height squared (kg/m^2^).

In order to explore differences between patients with great initial glucotoxicity and those with little initial glycemic burden, we separated these patients into two distinct subgroups: baseline HbA1c > 7.0% or ≤7.0%.

### 2.4. Statistical Analyses 

Statistical analysis was performed using SPSS version 22.0 for Windows (SPSS Inc., Chicago, IL, USA). Data were expressed as percentages, mean ± standard deviation. We compared glycemic burden and other covariates between patients, with and without treatment failure, using the chi-square test for categorical variables and independent t-test for continuous variables. A multivariable forward Cox proportional hazards regression analysis was performed for treatment failure with glycemic burden. Survival curves for estimate glycemic durability in two cohorts: baseline HbA1c > 7.0% or ≤7.0% were illustrated using the Kaplan–Meier method. Multivariable forward Cox proportional hazards regression analysis was performed separately, again, in these two different cohorts to investigate whether different determinants existed. MATLAB^®^ R2020b, a programming platform designed specifically for numeric computing that was popular in engineers and scientists, was used to automatically calculate glycemic burden (including AUC’ and AUC”) and durability length. A difference was considered significant at *p* < 0.05.

## 3. Results

### 3.1. Patients Baseline Characteristics

In the current study, we finally recruited 1048 newly-diagnosed drug-naïve T2DM patients. There were 291 patients with treatment failure, and their stable glycemic period lasted 26.8 ± 21.1 months; on the other hand, 757 patients without treatment failure were found with stable glycemic period lasted 45.1 ± 31.8 months. A comparison of the clinical characteristics among the participants, with and without treatment failure, is shown in Table 1. Glycemic burden, no matter AUC’ or AUC” was significantly more severe in patients with treatment failure (7.3 ± 12.7 vs. 3.4 ± 8.9%/90 days; 3.5 ± 3.0 vs. 1.9 ± 3.0%/90 days). Baseline HbA1c, TG, and total cholesterol were higher in treatment failure subgroup. In contrast, younger age was found in treatment failure subgroup. They also had greater chance to be prescribed SU, more categories of OAD, and more initially prescribed insulin.

### 3.2. Determinants of Treatment Failure in all Study Patients

Table 2 displayed determinants of treatment failure using forward Cox proportional hazards regression analysis. After adjusting age, gender, education history, SBP, DBP, BMI, initial HbA1C > 7.0%, glycemic burden (AUC’ and AUC’’), triglyceride, total cholesterol, HDL-cholesterol, LDL-cholesterol, eGFR, ALT, initial hospitalization, initially prescribed OAD (biguanides, TZD, DPP4i, GLP1RA, SU, glinides, AGIs, SGLT2i), initially prescribed OAD (categories), and initially prescribed insulin, we found that young age (*p* < 0.001), elementary school or uneducated (vs. high school, *p* = 0.020; vs. university or higher, *p* = 0.001), baseline HbA1c (*p* < 0.001), high glycemic burden AUC’ (per 1%/90 days; HR, 1.027; 95% CI, 1.019 to 1.035; *p* < 0.001), SU use (*p* = 0.036), AGIs use (*p* = 0.021), and no OAD use (vs. monotherapy, *p* = 0.002; vs. two combination therapy, *p* = 0.002; vs. three or more combination therapy, *p* = 0.001) were significantly associated with treatment failure. 

**Figure 3:**
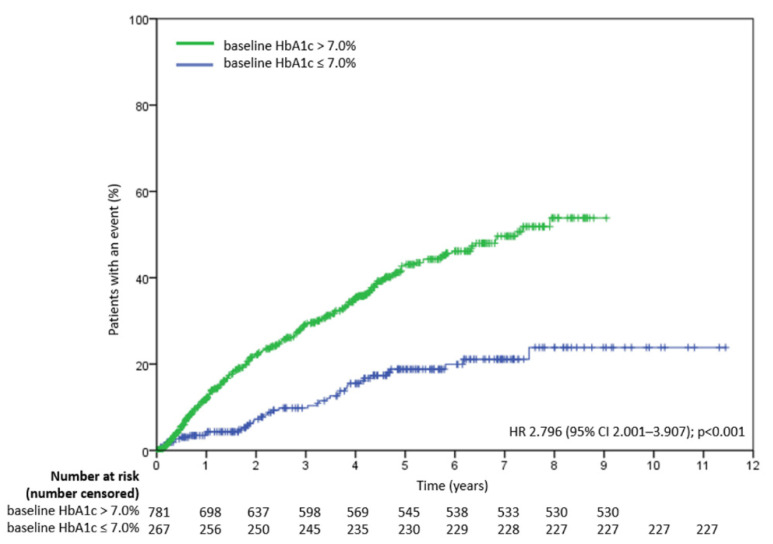
Time to treatment failure in two cohorts: baseline HbA1c > 7.0% and ≤7.0%. HR is based on Cox regression analysis. HR = hazard ratio.

Figure 3 displays time to treatment failure in two cohorts: baseline HbA1c > 7.0% and ≤7.0%. Baseline HbA1c > 7% was associated with treatment failure (vs. HbA1c ≤ 7.0 %; HR, 2.796; 95% CI, 2.001 to 3.907; *p* < 0.001). 

### 3.3. Determinants of Treatment Failure in Study Patients with Baseline HbA1c > 7.0%

Table 3 displayed determinants of treatment failure using forward Cox proportional hazards regression analysis in study patients with baseline HbA1c > 7.0% (n = 781). In patients with baseline HbA1c > 7.0%, after multivariable adjustment, young age (*p* < 0.001), elementary school or uneducated (vs. university or higher, *p* = 0.007), high glycemic burden AUC’ (per 1%/90 days; HR, 1.026; 95% CI, 1.018 to 1.034; *p* < 0.001), AGIs use (*p* = 0.048), and baseline HbA1c (per 1%; HR, 1.130; 95% CI, 1.058 to 1.171; *p* < 0.001) were significantly associated with treatment failure in study patients with baseline HbA1c > 7.0%.

### 3.4. Determinants of Treatment Failure in Study Patients with Baseline HbA1c ≤ 7.0%

Table 4 displayed determinants of treatment failure using forward Cox proportional hazards regression analysis in study patients with baseline HbA1c ≤ 7.0% (*n* = 267). In patients with baseline HbA1c ≤ 7.0%, after multivariable adjustment, young age (*p* = 0.001), low SBP (*p* = 0.041), high glycemic burden AUC” (per 1%/90 days; HR, 1.128; 95% CI, 1.016 to 1.253; *p* = 0.024), high TG (*p* = 0.001), low HDL-cholesterol (*p* = 0.033), low ALT (*p* = 0.044), and no OAD use (vs. monotherapy, *p* < 0.001; vs. two combination therapy, *p* < 0.001) were significantly associated with treatment failure in study patients with baseline HbA1c ≤ 7.0%.

## 4. Discussion

In the present study, we found that glycemic burden, assessed by AUC, was associated with future long-term glycemic durability in real-world clinical care of newly-diagnosed T2DM patients. Furthermore, “metabolic memory” phenomenon was also observed, as initial glucotoxicity (AUC’) had a greater impact on further treatment failure rate than glycemic burden after initial hyperglycemia resolution (AUC”). Finally, despite the divergence of durability and different determinants, in patients whose baseline HbA1c > 7.0% and ≤7.0%, glycemic burden still played an important role in both cohorts.

Although clinicians have been interested in heterogeneity of glycemic progression in T2DM patients [34], few studies correlated it with glycemic insult per se. Post hoc analyses of Treatment Option for Type 2 Diabetes in Adolescents and Youth (TODAY) trial and DISCOVERing Treatment Reality of Type 2 Diabetes in Real World Setting (DISCOVER) studies revealed that being unable to attain good glucose control on metformin or second-line glucose-lowering therapy, respectively, appeared predictive of loss of glycemic control in the following 2 to 3 years [35,36]. However, neither of them quantified hyperglycemia burden and displayed its correlation with the duration of stable glycemic control as our study did. The precisely calculated HbA1c AUC could stand for glycemic burden and glucotoxicity. Moreover, we did not define durability according to the HbA1c value at a limited timepoint, but we kept tracking the HbA1c trend until it exceeded treatment failure value, which was theoretically more intuitive on clinical practice. 

As mentioned before, we divided AUC into AUC’ and AUC”. Both compartments were associated with treatment failure in univariate analyses, but only AUC’, the most primitive burden before newly-diagnosed hyperglycemia resolved, remained significant in multivariate analysis. This indirectly confirmed our hypothesis that “legacy effect” or “metabolic memory” didn’t only exist in macrovascular complications but also in the durability of pancreatic β-cells. Notice that using Kaplan–Meier method revealed divergent treatment failure curves of two subgroups (baseline HbA1c > 7.0% or ≤7.0%) verified this concept again, as scarcely had the latter group been threatened by severe glucotoxicity initially, and its AUC’ could be considered to be “0”, as it also had a significant lower failure rate. The possible mechanism might be that transient hyperglycemia induced long-lasting activating epigenetic changes through adjusting histone methylation in affected cells [37,38]. 

In this study, we also reconfirmed some factors that were proved to be related to glycemic durability before. Brown et al. found not only higher baseline HbA1c or younger age but also delayed starting metformin monotherapy just after T2DM was diagnosed could predict higher secondary failure rate [22], and our study extended the same result to other initial OAD combinations. Concerning initial OAD choice, our participants who were prescribed SU or AGI appeared to have a greater chance of hyperglycemia relapse. Though several studies showed the efficacy of SU or AGI comparable with Metformin in short and medium term, neither of these two drugs improve glycemic control via increasing insulin sensitivity, and this was responsible for inferior glycemic durability of SU to metformin and TZD in a long term ADOPT trial [18,39,40]. Whether T2DM patients’ educational level had an impact on glycemic control or not were inconclusive in previous studies [41,42]. Our study revealed patients who were uneducated or received elementary school education had higher failure risk than those who received higher education, and the underlying mechanism could be that healthy lifestyles and exercise habits were more prevalent in better educated T2DM patients [43].

Glycemic burden and age were the only two determinants which simultaneously affected durability in two subgroups classified by baseline HbA1c (>7.0% or ≤7.0%). Notice that all AUC’ of the latter cohort were a constant “0”, and AUC” replaced AUC’ as a determinant in this situation, which implied that small but slowly accumulative glycemic burden would still increase treatment failure risk in the future. Other original determinants influenced durability, respectively, in two subgroups without overlapping. Lower baseline HbA1c value, higher education level than high school, and not using AGI initially got lower risk of hyperglycemia progression in baseline HbA1c > 7.0% subgroup. These factors were probably no longer determinants of durability in the other subgroup because narrow extent of baseline HbA1c (6.5% to 7.0%), little initial glycemic insult, and less frequencies use of AGI respectively decreased the impact of these factors. On the contrary, no OAD use initially still played a part in the baseline HbA1c ≤ 7.0% subgroup but not in the other. The possible explanation of this discrepancy could be that some patients were prescribed insulin instead of any OAD in baseline HbA1c > 7.0% subgroup, but this would hardly happen in baseline HbA1c ≤ 7.0% subgroup. Several new determinants associated with higher treatment failure rate emerged in baseline HbA1c ≤ 7.0% subgroup. Some were reasonable, such as lower HDL and higher TG, and were proved to be a sensitive index of insulin resistance in nondiabetic obese patients [44]. Some were difficult to interpret, such as lower SBP and lower ALT. As multifactorial intervention, including hypertension therapy in diabetic patients, is fundamental, it is possible that a physician had put major efforts in controlling other risk factors (such as lowering SBP) and, therefore, neglected to maintain further glycemic stability in these initially glycemic relative well-controlled patients [45]. For ALT, the values reported are within the reference range in both groups. It is possible that it is only a stochastic finding.

There are several limitations to this study. First, this is a retrospective observational study, so we could not define the causal relationship between glycemic burden and glycemic durability. It is possible that AUC’ or AUC” is only an early omen of treatment failure, and whether the interventions that try to reduce initial glycemic burden would guarantee longer good glycemic control is uncertain. The Restoring Insulin Secretion (RISE) Adult Medication Study failed to produce persistent benefits of β-cell function after initial treatment withdrawal [46]. Although the Verify study represented the success of early treatment intensification, we could not attribute the benefit to decreasing initial glucotoxicity unless further post-hoc analysis or new randomized control trials developed for this goal [19]. Second, some severe hyperglycemia patients were excluded due to failure of achieving treatment goal value. As a result of our study design, these patients were abandoned due to difficulty of defining their glycemic durability. Thirdly, we could not estimate the impact of non-medical treatments (such as dietary and physical activities) on glycemic durability in this study, due to a lack of completed records in our database. Finally, due to the restrictions of EMR and format of DSCP, disease duration of newly-diagnosed T2DM patients was defined to within 1 year instead of a shorter period, and it might underestimate initial glycemic burden AUC’. However, by means of only retaining drug-naïve patients, most patients with severe initial glucotoxicity would not delay too long before they were recruited in this study. 

In conclusion, this medium-sized, well-controlled, real-world cohort study verified that the secondary treatment failure of newly-diagnosed T2DM patients, namely glycemic durability, were negatively associated with greater glucotoxicity, defined by glycemic burden AUC, especially the burden before initial hyperglycemia resolved.

## Figures and Tables

**Table 1:** Comparison of clinical characteristics among participants, according to without or with treatment failure.

Characteristics	Without Treatment Failure (*n* = 757)	With Treatment Failure (*n* = 291)	*p*
Age (year)	56.8 ± 12.6	53.2 ± 12.1	<0.001
Male gender (%)	54.2	57.7	0.298
Education history			0.058
Elementary school or uneducated (%)	25.9	28.3	
High school (%)	47.7	52.4	
University or higher (%)	26.4	19.3	
SBP (mmHg)	134.3 ± 18.1	132.0 ± 20.7	0.107
DBP (mmHg)	79.9 ± 11.9	80.2 ± 12.5	0.699
BMI (kg/m^2^)	26.2 ± 4.6	26.6 ± 4.8	0.198
Glycemic burden AUC’ (%/90 days)	3.4 ± 8.9	7.3 ± 12.7	<0.001
Glycemic burden AUC’’(%/90 days)	1.9 ± 3.0	3.5 ± 3.0	<0.001
Laboratory parameters			
Baseline HbA1c (%)	9.3 ± 2.6	10.7 ± 2.8	<0.001
Triglyceride (mg/dL)	151.5 ± 103.3	181.9 ± 144.8	0.001
Total cholesterol (mg/dL)	188.8 ± 44.5	196.5 ± 48.5	0.015
HDL-cholesterol (mg/dL)	44.0 ± 12.3	42.9 ± 12.0	0.171
LDL-cholesterol (mg/dL)	115.1 ± 36.6	119.1 ± 41.8	0.153
eGFR (mL/min/1.73 m^2^)	95.4 ± 30.7	100.9 ± 43.6	0.024
ALT (u/L)	40.4 ± 35.1	42.7 ± 41.5	0.385
Initial insulin pump (%)	7.7	16.8	<0.001
Initially prescribed OAD			
Biguanides (%)	87.2	85.9	0.584
TZD (%)	4.9	5.8	0.531
DPP4i (%)	46.2	44.3	0.579
GLP1RA (%)	0.1	0.0	0.535
SU (%)	35.4	53.6	<0.001
Glinides (%)	3.3	3.4	0.914
AGI (%)	1.2	2.7	0.073
SGLT2i (%)	0.3	0.0	0.380
Initially prescribed OAD (category number)			<0.001
No OAD (%)			
Monotherapy (%)	5.9	9.6	
Two combination therapy (%)	33.2	20.3	
Three or more combination therapy (%)	38.6	38.5	
Initially prescribed insulin use	22.3	31.6	0.017
No insulin (%)	86.5	79.7	
Basal insulin (%)	11.8	16.8	
Basal and pre-prandial insulin (%)	1.7	3.4	
Stable glycemic period (months)	45.1 ± 31.8	26.8 ± 21.1	<0.001

Abbreviations. SBP, systolic blood pressure; DBP, diastolic blood pressure; BMI, body mass index; HbA_1c_, glycated hemoglobin; AUC, area under the curve; HDL, high-density lipoprotein; LDL, low-density lipoprotein; eGFR, estimated glomerular filtration rate; ALT, alanine aminotransferase; OAD, oral antidiabetic; TZD, thiazolidinediones; DPP4i, dipeptidyl peptidase-4 inhibitor; GLP1RA, glucagon-like peptide-1 receptor agonist; SU, sulfonylureas; AGI, α-Glucosidase inhibitor; SGLT2i, sodium-glucose cotransporter 2 inhibitor. Treatment failure was defined as HbA_1c_ rebounded to treatment failure value (HbA1c ≥ 8.0%). Glycemic burden AUC was defined to the area above a burden value (6.5%) but under the HbA1c curve. AUC was then divided into 2 compartments: AUC’ represents the burden during run-in period; AUC” represents the burden during stable glycemic period.

**Table 2:** Determinants of glycemic burden and other clinical characteristics with treatment failure in all study participants (*n* = 1048) using multivariable forward Cox proportional hazards analysis.

Variables	Multivariable (Forward)	
	HR (95% CI)	*p*
Age (per 1 year)	0.959 (0.947, 0.971)	<0.001
Education history		0.005
Elementary school or uneducated	Reference	
High school	0.681 (0.492, 0.942)	0.020
University or higher	0.495 (0.324, 0.754)	0.001
Glycemic burden AUC’ (per 1 %/90 days)	1.027 (1.019, 1.035)	<0.001
Laboratory parameters		
Baseline HbA1c (%)	1.118 (1. 065, 1. 174)	<0.001
Initially prescribed OAD		
SU (vs. no use SU)	1.459 (1.026, 2.076)	0.036
AGI (vs. no use AGI)	2.496 (1.150, 5.422)	0.021
Initially prescribed OAD (categories)		0.004
No OAD	Reference	
Monotherapy	0.474(0.294, 0.763)	0.002
Two combination therapy	0.457(0.281, 0.743)	0.002
Three or more combination therapy	0.370(0.211, 0.650)	0.001

Values are expressed as hazard ratio (HR) and 95% confidence interval (CI). Adjusting for age, gender, education history, SBP, DBP, BMI, initial HbA1C > 7.0%, glycemic burden (AUC’ and AUC’’), triglyceride, total cholesterol, HDL-cholesterol, LDL-cholesterol, eGFR, ALT, initial hospitalization, initially prescribed OAD (biguanides, TZD, DPP4i, GLP1RA, SU, glinides, AGI, SGLT2i), initially prescribed OAD (category number), and initially prescribed insulin. Abbreviations are the same as Table 1.

**Table 3:** Determinants of glycemic burden and other clinical characteristics with treatment failure in initial HbA_1c_ > 7.0% participants (*n* = 781) using multivariable forward Cox proportional hazards analysis.

Variables	Multivariable (Forward)	
	HR (95% CI)	*p*
Age (per 1 year)	0.965 (0.952, 0.978)	<0.001
Education history		0.026
Elementary school or uneducated	Reference	
High school	0.721 (0.504, 1.030)	0.073
University or higher	0.531 (0.335, 0.841)	0.007
Glycemic burden AUC’ (per 1 %/90 days)	1.026 (1.018, 1.034)	<0.001
Laboratory parameters		
Baseline HbA1c (%)	1.130 (1.058, 1.171)	<0.001
Initially prescribed OAD		
AGIs (vs. no use AGIs)	2.161 (1.007, 4.638)	0.048

Values expressed as hazard ratio (HR) and 95% confidence interval (CI). Adjusting for age, gender, education history, SBP, DBP, BMI, glycemic burden (AUC’ and AUC’’), triglyceride, total cholesterol, HDL-cholesterol, LDL-cholesterol, eGFR, ALT, initial hospitalization, initially prescribed OAD (biguanides, TZD, DPP4i, GLP1RA, SU, glinides, AGI, SGLT2i), initially prescribed OAD (category number), initially prescribed insulin, and HbA1C_Max. Abbreviations are the same as Table 1.

**Table 4:** Determinants of glycemic burden and other clinical characteristics with treatment failure in initial HbA_1c_ ≤ 7.0% participants (*n* = 267) using multivariable forward Cox proportional hazards analysis.

Variables	Multivariate (Forward)	
	Unstandardized Coefficient *β* (95% CI)	*p*
Age (per 1 year)	0.938 (0.903, 0.975)	0.001
SBP (per 1 mmHg)	0.976 (0.953, 0.999)	0.041
Glycemic burden AUC’’ (per 1 %/90 days)	1.128 (1.016, 1.253)	0.024
Laboratory parameters		
Triglyceride (per 1 mg/dL)	1.004 (1.002, 1.006)	0.001
HDL-cholesterol (per 1 mg/dL)	0.960 (0.924, 0.997)	0.033
ALT (per 1 u/L)	0.977 (0.955, 0.999)	0.044
Initially prescribed OAD (categories)		<0.001
No OAD (%)	Reference	
Monotherapy (%)	0.164 (0.072, 0.371)	<0.001
Two combination therapy (%)	0.123 (0.042, 0.365)	<0.001
Three or more combination therapy (%)	1.256 (0.090, 17.486)	0.865

Values are expressed as hazard ratio (HR) and 95% confidence interval (CI). Adjusting for age, gender, education history, SBP, DBP, BMI, glycemic burden AUC’’, triglyceride, total cholesterol, HDL-cholesterol, LDL-cholesterol, eGFR, ALT, initially prescribed OAD (biguanides, TZD, DPP4i, GLP1RA, SU, glinides, AGI, SGLT2i), initially prescribed OAD (categories), and initially prescribed insulin. Abbreviations are the same as Table 1.

## Data Availability

The data underlying this study is from Kaohsiung Medical University Hospital and Kaohsiung Municipal Siaogang Hospital. Due to restrictions placed on the data by the Institutional Review Board of Kaohsiung Medical University Hospital, the minimal data set cannot be made publicly available.
